# Supplementary material for: Modeling CRISPR-Cas13d on-target and off-target effects using machine learning approaches
Source: Nat Commun. 2023 Feb 10;14:752. doi: 10.1038/s41467-023-36316-3 (PMC9912244; doi:10.1038/s41467-023-36316-3)
Supplement: Supplementary file 1 — Supplementary Information [file 41467_2023_36316_MOESM1_ESM.pdf]

Modeling CRISPR-Cas13d on-target and off-target effects using machine learning approaches

Xiaolong Cheng[1,2,6], Zexu Li[3,6], Ruocheng Shan[1,4], Zihan Li[3], Shengnan Wang[3], Wenchang Zhao[3], Han Zhang[3], Lumen Chao[1,2], Jian Peng[5], Teng Fei[3*], Wei Li[1,2*]

* Correspondence email: wli2@childrensnational.org; feiteng@mail.neu.edu.cn

Supplementary

Supplementary Fig. 1 Summary of CRISPR-Cas13d screens.

(a) The scores of essential genes, non-essential genes and other genes in A375 RNAi screens and CRISPR screens in DepMap. CRISPR scores are calculated using CERES, while RNAi scores are calculated using DEMETER in DepMap. (**b**) The RRA score distribution of negative selection (day 33 vs day 5), reported by the MAGeCK algorithm. (**c**) The overall enrichment of guides targeting essential genes in the ranking of all the guides, estimated by Gene Set Enrichment Analysis (GSEA). ES: enrichment score; NES: normalized enrichment score. The p-value is calculated by permutation in GSEA.

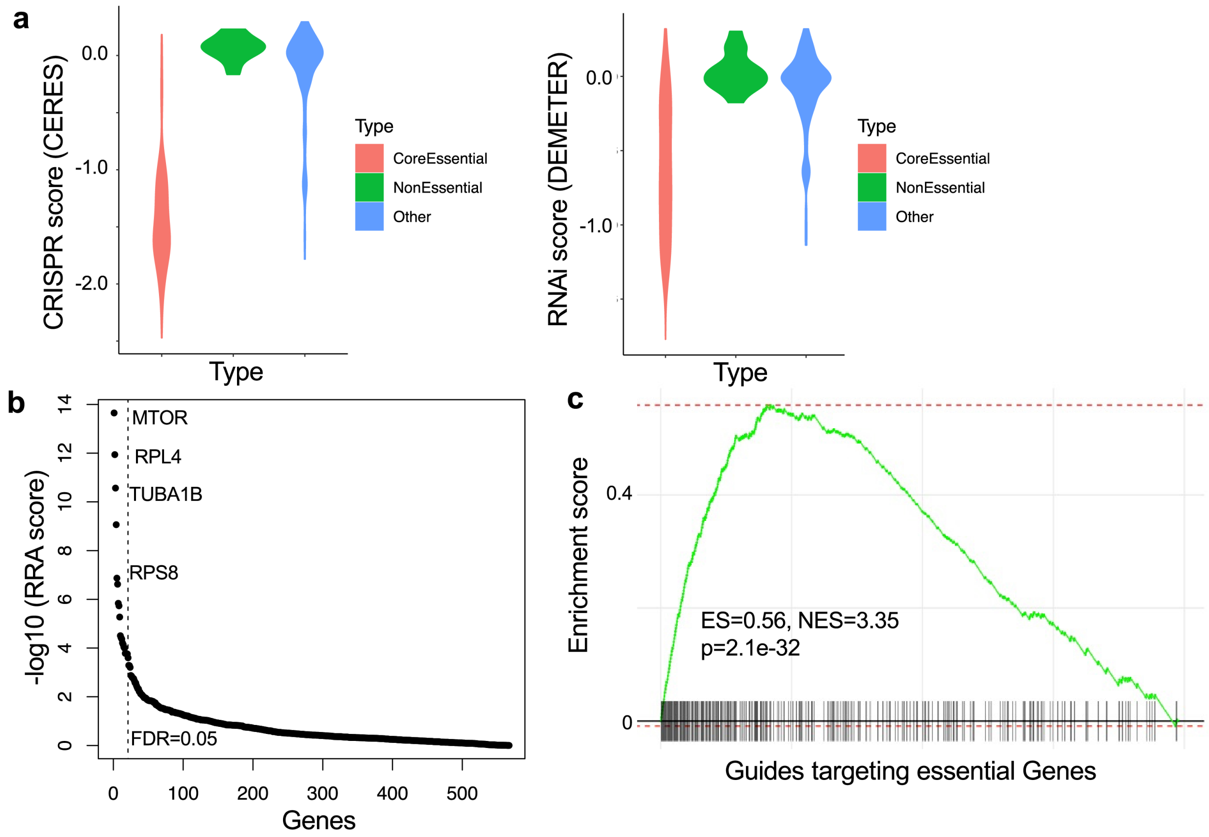

Supplementary Fig. 2 sgRNA secondary structure is beneficial to model precision. (**a**) Scatterplot shows the relationship between MFE and LFC. (**b**) Average precision comparison with or without sgRNA secondary structure. n=5 folds used in cross-validation. The two-sided independent t-test is used for analysis. (**c-g**) Precision-recall curves from 5-fold cross-validation. The top, mid-line and bottom of the boxplot (**b**) represents the upper quartile (Q3), median, and lower quartile (Q1), respectively. The ends of the whiskers represent the minimum and maximum values in the data set. This definition applies to all boxplots in other supplementary figures.

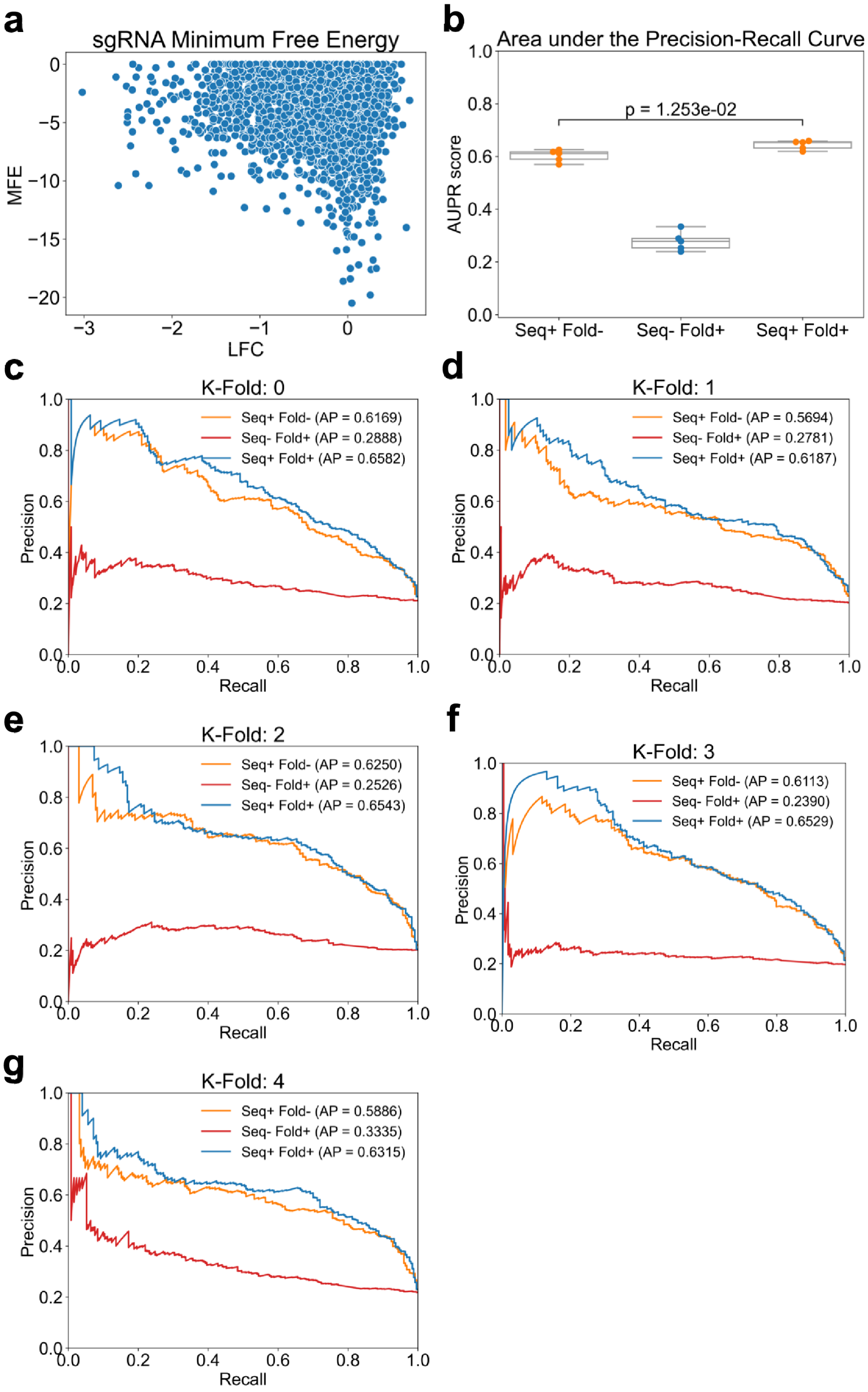

Supplementary Fig. 3 DeepCas13 can distinguish between effective and invalid sgRNAs.

(**a**) ROC curves of the 1st fold of validation data and the AUC scores are shown in the legend. (**b-e**) ROC curves of the 2nd-5th fold of validation data and the AUC scores are shown in the legend.

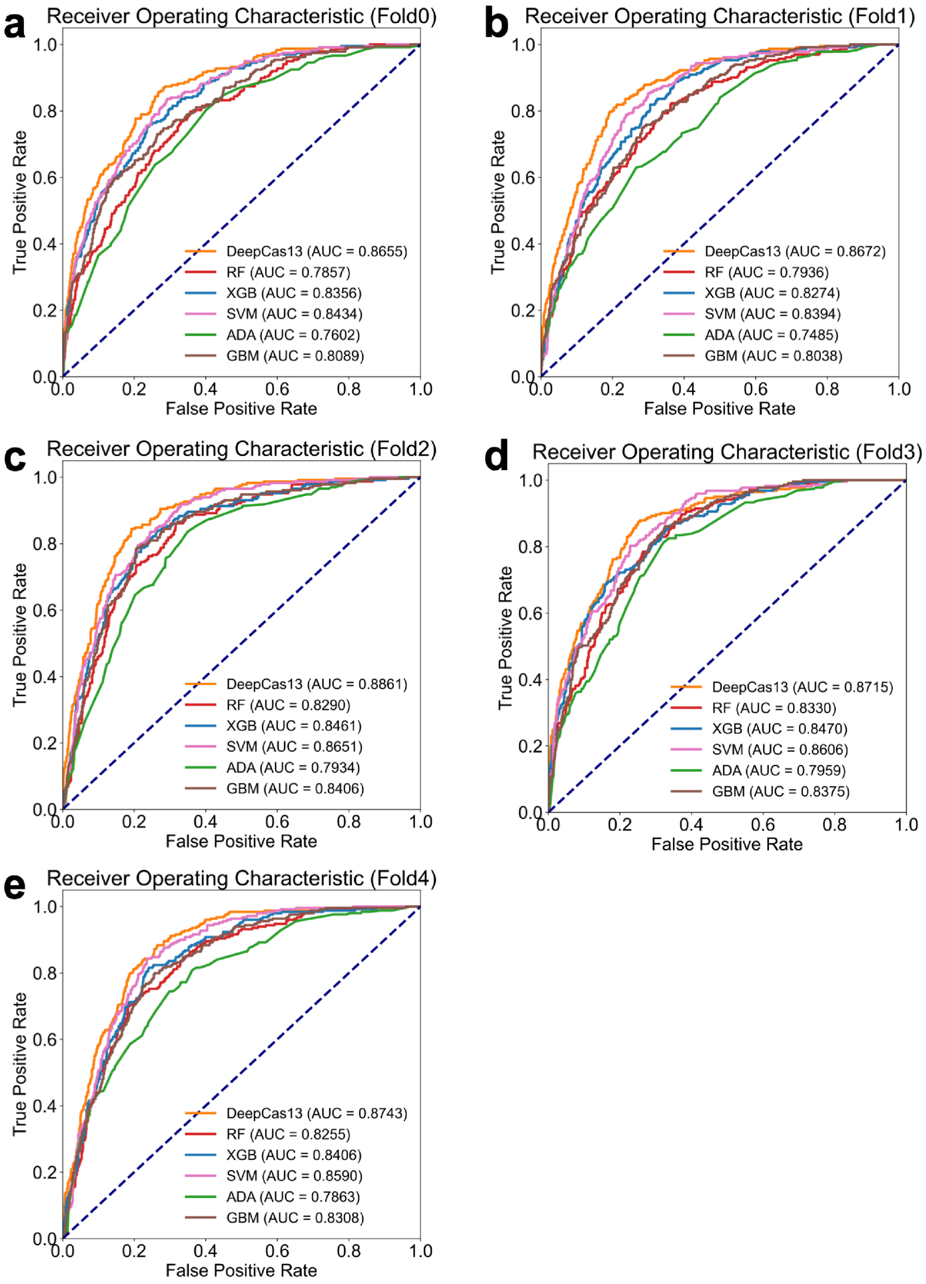

Supplementary Fig. 4 DeepCas13 can reach high prediction precision.

(**a**) PRC curves of the 1st fold of validation data and the AUPR scores are shown in the legend. (**b-e**) PRC curves of the 2nd-5th fold of validation data and the AUPR scores are shown in the legend.

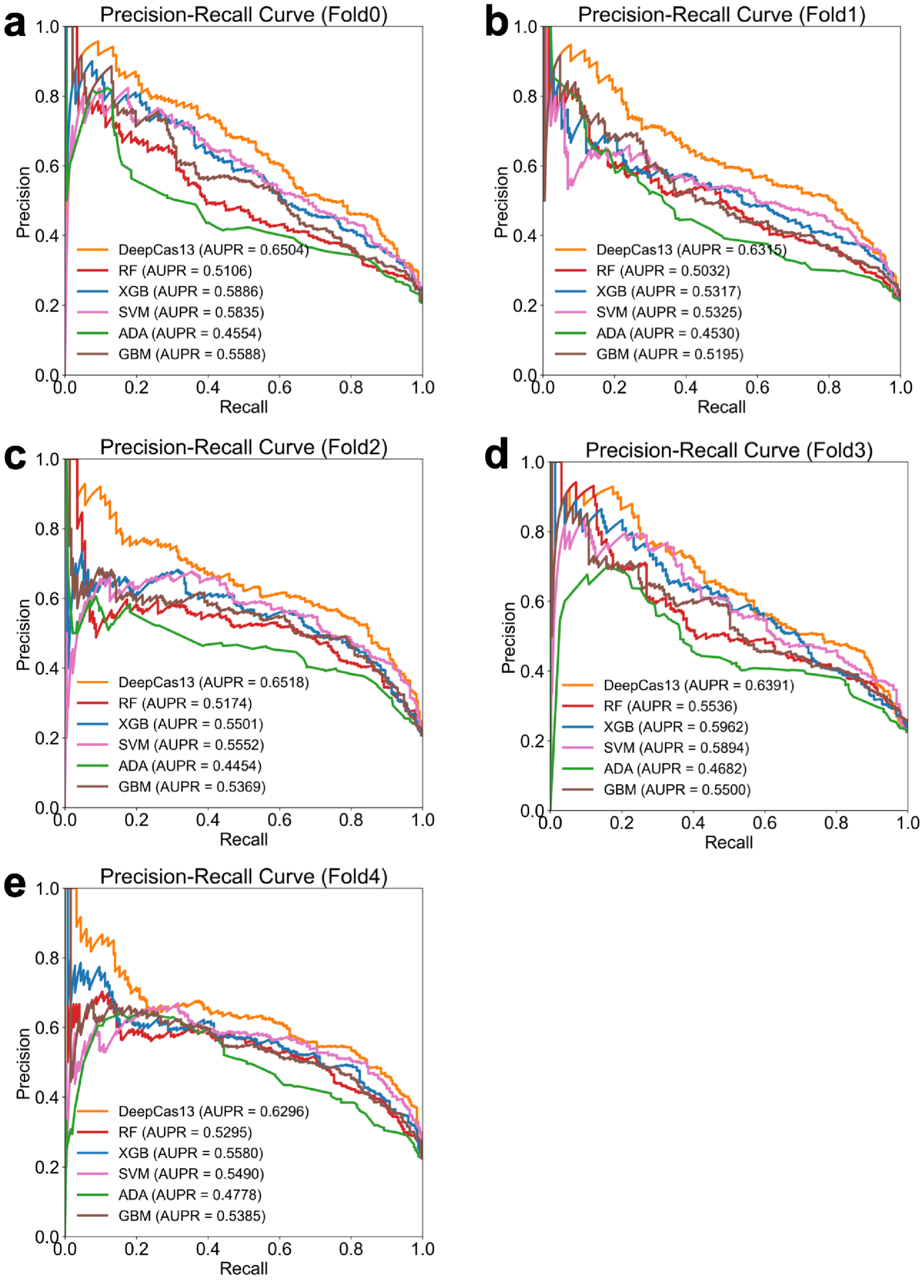

Supplementary Fig. 5 Leave-one-dataset-out evaluation.

(**a**) ROC curve comparison for the public Cas13d proliferation dataset. Orange curve means both FACS sorting data and proliferation data are used for the training. Red curve means only FACS sorting data is used for the training. Blue curve shows the performance of the existing tool. (**b**) PRC curve comparison for the public Cas13d proliferation dataset. (**c**) ROC curve comparison for our Cas13d proliferation dataset. (**d**) PRC curve comparison for our Cas13d proliferation dataset. (**e**) boxplot shows the Integrated Gradients values at each position. n=5,726 guides. (**f**) Base preference based on Integrated Gradients technique.

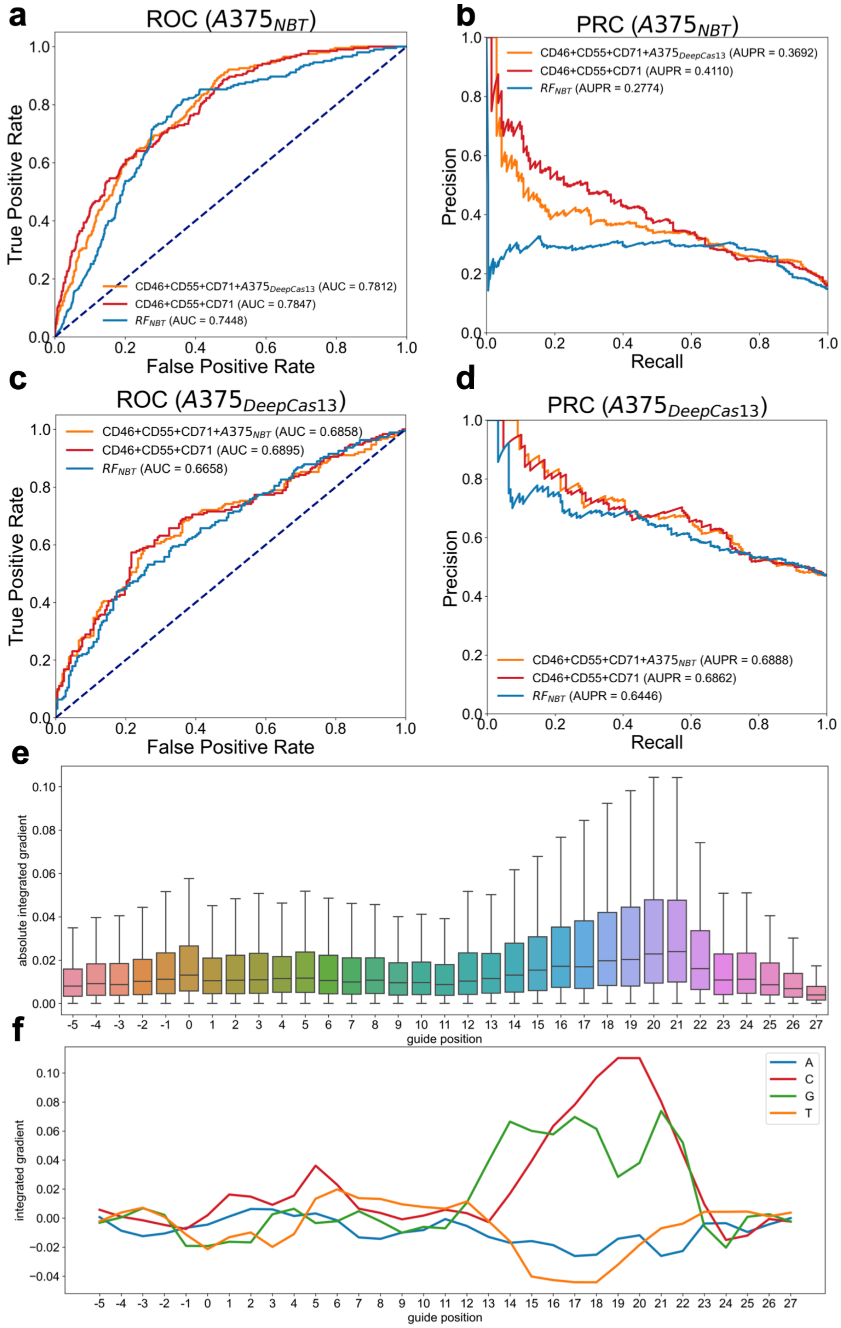

Supplementary Fig. 6 Off-target viability effect analysis.

(**a**) The predicted off-target viability score (using non-essential genes) on guides that showed strong on-target knockdown ("Dropped" guides) of target genes in FACS-based Cas13d screens vs. other guides. The "Dropped" guides are guides that demonstrated strongest dropout in GFP+ population. n=1789, 1925 and 2012 guides targeting CD46, CD55 and CD71 respectively. The p-values are calculated by k-s test. (**b**) The true positive rate (in identifying known essential genes) with different FDR cutoff using different controls: no controls or non-essential controls. The A375 screens in our study was used. Non-targeting control analysis was not included as the library did not contain non-targeting controls. (**c**) The false positive rate (in identifying non-essential genes as significant).

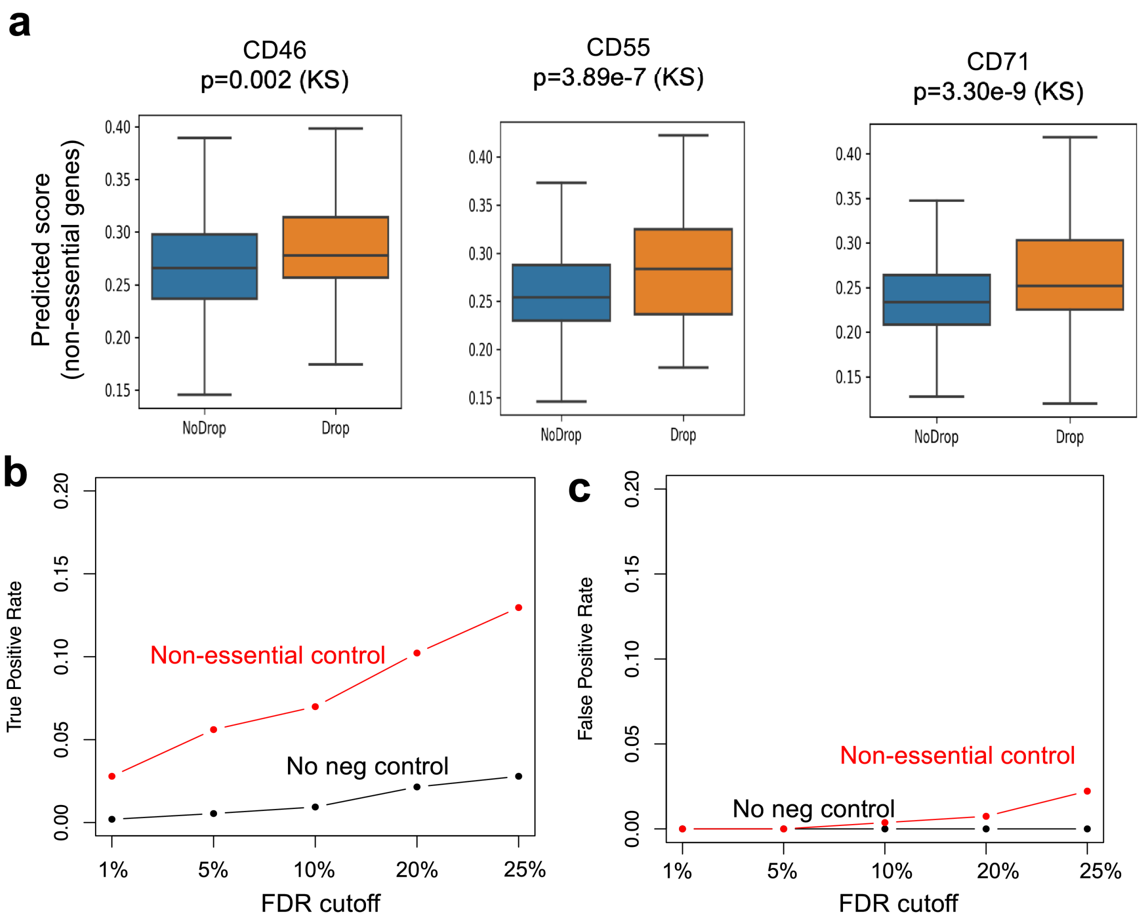

Supplementary Fig. 7 lncRNA screening analysis.

(**a**) The gene set enrichment analysis (GSEA) of guides targeting essential genes, in all the guides in the screen. Guides are ranked by their negative selection in A549 cells. (**b**) The distribution of RRA scores, measured in the screen, of lncRNAs with high (or low) expressions in A549 cells. **(c-d)** The survival analysis of NEAT1 and SNHG29 expression in TCGA melanoma cohort (Skin Cutaneous Melanoma). The progression-free survival of NEAT1 and overall survival of SNHG29 is used. The analysis is performed in TANRIC platform(*53*). The *p* value is calculated using linear regression (LR). n=89 lncRNAs. The p-value is calculated by permutation in GSEA and calculated by log rank test in survival curve. (**e**) The RRA scores of essential genes across two different cell lines. Error band shows the 95% confidence interval for the regression estimate. The p-value is two-sided and calculated by a test of the null hypothesis that the distributions underlying the samples are uncorrelated and normally distributed. PCC: Pearson Correlation Coefficient (**f**) The sgRNA changes of two lncRNAs (CD27-AS1 and CYTOR) across two different cell lines.

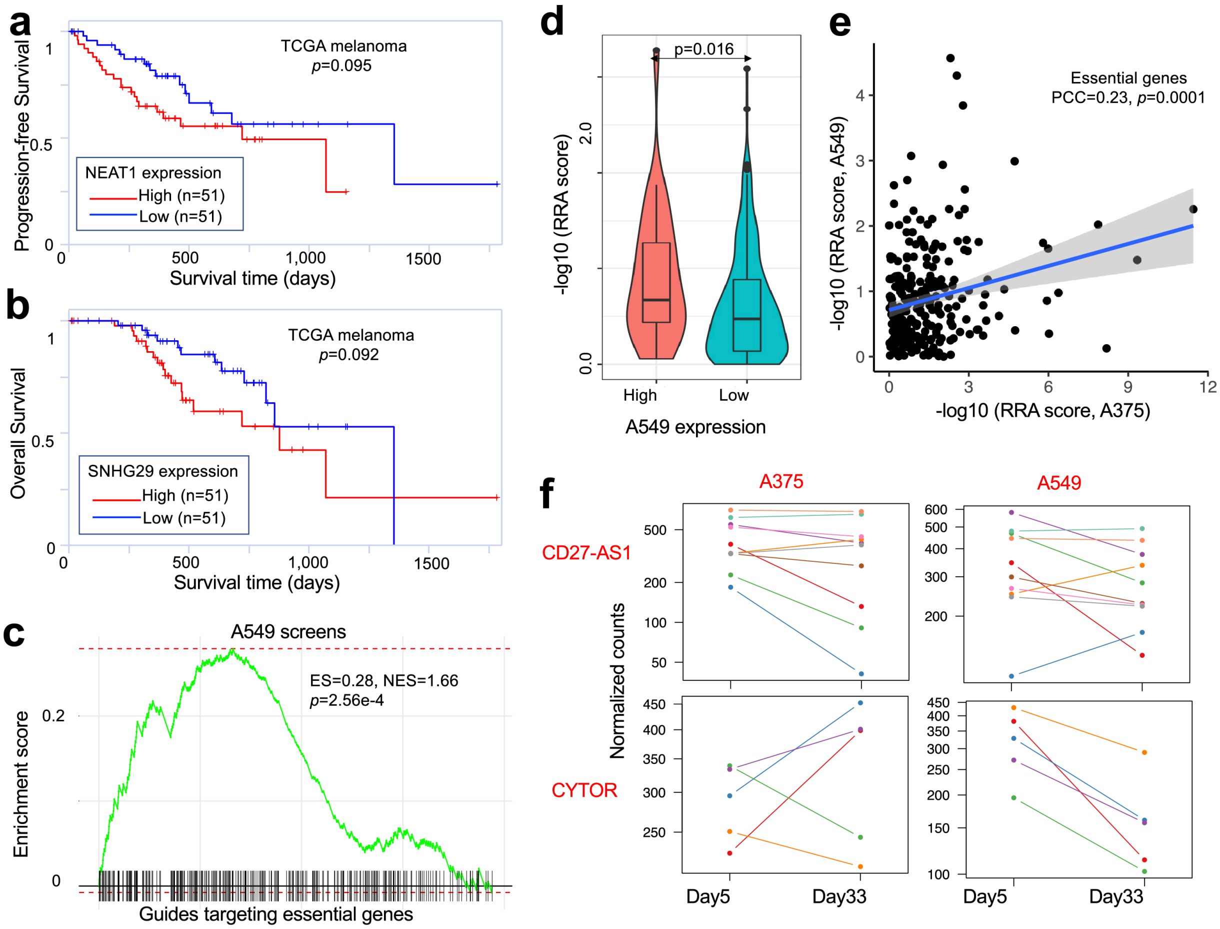

Supplementary Fig. 8 Analysis of validation experiments.

(**a**) LFC distribution of the top 15% predicted sgRNA for each group. n=948, 369 and 107 guides targeting mRNA, lncRNA and circRNA respectively. (**b**) LFC distribution of the top 20% predicted sgRNA for each group. n=1264, 492 and 143 guides targeting mRNA, lncRNA and circRNA respectively. (**c**) LFC distribution of sgRNAs targeting coding genes, lncRNAs, circRNAs and control sgRNAs. (**d**) ECDF distribution of sgRNAs for each group. (**e**) LFC distribution of high and low predicted sgRNAs for individual lncRNA. *H* means sgRNA with high predicted score while *L* means sgRNA with low predicted score. n=342, 346, 373, 370, 194 and 370 guides in $H_{DeepCas13}$, H_{RFNBT}, $L_{DeepCas13}$, L_{RFNBT}, H_{both}, L_{both} group respectively. (**f**) LFC distribution of high and low predicted sgRNAs for individual circRNA. n=40, 21, 250, 280, 4 and 252 guides in $H_{DeepCas13}$, H_{RFNBT}, $L_{DeepCas13}$, L_{RFNBT}, H_{both}, L_{both} group respectively. (**g**) sgRNAs with low qPCR performance. n=3 independent qPCR experiments. Error bar shows the 95% confidence interval.

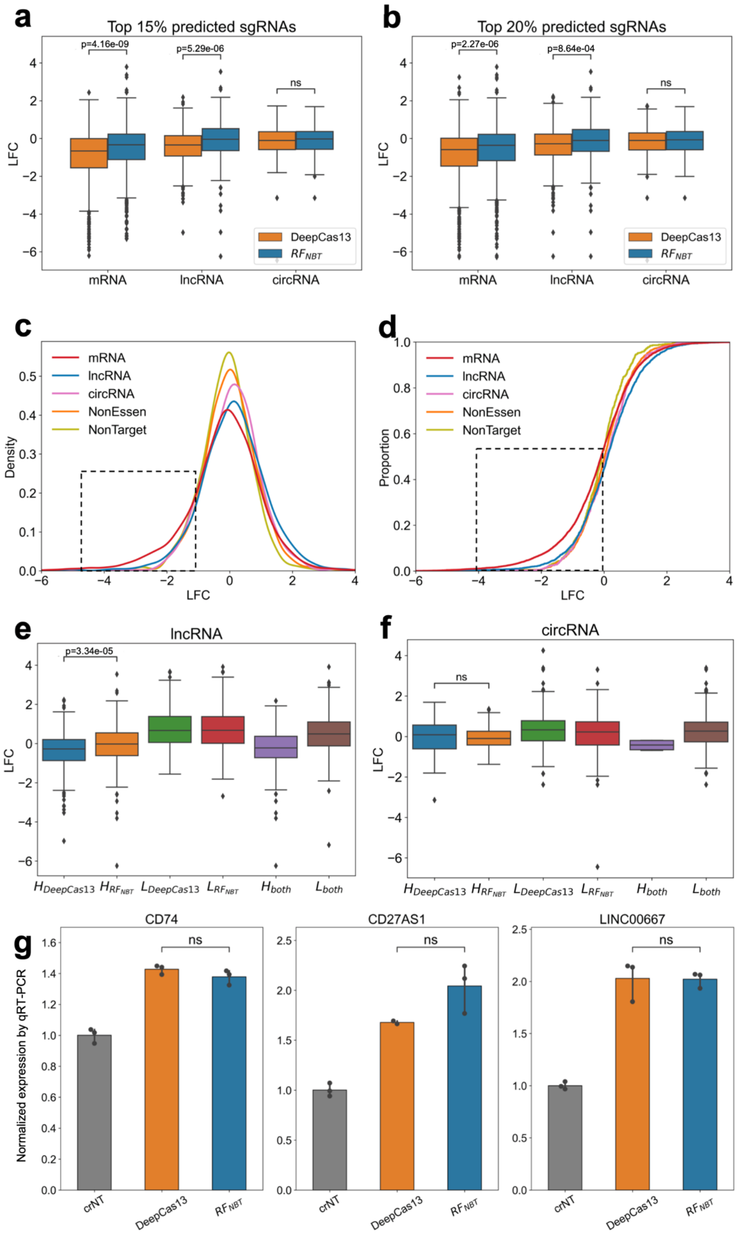

Supplementary Table 1

The QC metrics reported by the MAGeCK algorithm.

Sample	Reads	Mapped	Percentage	Total sgRNAs	Reads per sgRNA	Zero counts	Gini Index
A375_Day5_R1	6,923,422	5,745,864	0.8299	10,829	530.60	1	0.04066
A375_Day5_R2	4,540,478	3,772,583	0.8309	10,829	348.38	1	0.0451
A375_Day35_R1	4,944,792	4,127,582	0.8347	10,829	381.16	2	0.05567
A375_Day35_R2	6,114,984	5,033,558	0.8232	10,829	464.82	3	0.05655

Supplementary Table 2

Summary of training data used in this study.

Dataset	sgRNA number	Target	Source
Cas13d A375 pooled screens data	10,830	essential/non-essential genes, lncRNAs	this study
Cas13d tiling screens data	5,726	CD46, CD55, CD71	PMID: 32518401
Cas13d A375 pooled screens data	1,398	essential genes	PMID: 32518401
Cas13d circRNA screens data	3,800	circRNAs	PMID: 33288960
Cas13d circRNA screens data	845	circRNAs	PMID: 33478577
