# Supplementary material for: Modeling CRISPR-Cas13d on-target and off-target effects using machine learning approaches
Source: Nat Commun. 2023 Feb 10;14:752. doi: 10.1038/s41467-023-36316-3 (PMC9912244; doi:10.1038/s41467-023-36316-3)
Supplement: Supplementary file 2 — Description of Additional Supplementary Files [file 41467_2023_36316_MOESM2_ESM.pdf]

Description of Additional Supplementary Files

File Name: Supplementary Data 1
Description: Essential genes screening summary

File Name: Supplementary Data 2
Description: Cas13d tiling data features

File Name: Supplementary Data 3
Description: DeepCas13 training dataset

File Name: Supplementary Data 4
Description: qPCR primer
